# Perceptions about cancer and barriers towards cancer screening among ethnic minority women in a deprived area in Denmark – a qualitative study

**DOI:** 10.1186/s12889-020-09037-1

**Published:** 2020-06-12

**Authors:** Camilla Rahr Tatari, Berit Andersen, Trine Brogaard, Sara Koed Badre-Esfahani, Negin Jaafar, Pia Kirkegaard

**Affiliations:** 1 Department of Public Health Programmes, Randers Regional Hospital, Randers, Denmark; 2 Department of Clinical Medicine, Aarhus University, Aarhus, Denmark; 3 Laegerne i Gellerup, Brabrand, Denmark; 4 Department of Gynaecology and Obstetrics, Aarhus University Hospital, Aarhus, Denmark

**Keywords:** Mass screening, Early detection of Cancer, Participation, Non-participation, Healthcare disparities, Ethnic groups, Emigrants and immigrants, Qualitative research, Denmark

## Abstract

**Background:**

Screening programmes for cervical cancer, breast cancer and colorectal cancer have been implemented in many Western countries to reduce cancer incidence and mortality. Ethnic minority women are less likely to participate in cancer screening than the majority population. In worst case this can result in higher incidence rates, later diagnosis and treatment and ultimately inferior survival. In this paper we explored the perceptions about cancer and perceived barriers towards cancer screening participation among ethnic minority women in a deprived area in Denmark.

**Methods:**

Interview study with ethnic minority women in a deprived area in Denmark. The interviews were transcribed verbatim followed by an inductive content analysis.

**Results:**

Cancer was perceived as a deadly disease that could not be treated. Cancer screening was perceived as only relevant if the women had symptoms. Knowledge about cancer screening was fragmented, often due to inadequate Danish language skills and there was a general mistrust in the Danish healthcare system due to perceived low medical competences in Danish doctors. There was, however, a very positive and curious attitude regarding information about the Danish cancer screening programmes and a want for more information.

**Conclusion:**

Ethnic minority women did not have sufficient knowledge about cancer and the purpose of cancer screening. Perceptions about cancer screening were characterised by openness and the study showed positive and curious attitudes towards screening participation. The findings emphasise the importance of culturally adapted interventions for ethnic minority women in attempts to reduce inequality in screening participation.

## Background

Cancer screening can detect cancer or precancerous lesions before symptoms appear. Participation in cancer screening can improve treatment and reduce incidence and mortality [1–3]. Many Western countries have implemented cancer screening programmes for cervical cancer, breast cancer and colorectal cancer.

Inequalities in participation in all the cancer screening programmes can be considered a problem. In worst case this can result in higher incidence rates, later diagnosis and treatment, and ultimately inferior survival. Reducing inequalities in participation in cancer screening programmes are high on the global cancer agenda [4]. As a group, ethnic minority women in deprived areas are characterised by lower employment, lower disposable income and education, as well as higher incidence of some cancers compared to the native population [5, 6]. Studies from Denmark and other countries show that ethnic minority women are less likely to participate in screening programmes for cervical cancer [7–10], breast cancer [11, 12] and colorectal cancer [13, 14] than the native population. There may be different complex factors influencing ethnic minority women’s screening participation. The literature focuses on the influence of tradition, beliefs, religion and culture [15]. Reasons for non-participation among ethnic minority women may be poor knowledge about cancer and screening, discomfort with the screening methods and fatalistic beliefs about cancer [16–18].

A recent review of interventions to improve the screening participation across a range of conditions in ethnic minority groups found that most multifaceted interventions, group-education and media interventions were successful [19]. However, the review also emphasised that community-based interventions are particularly sensitive to context, and despite focused attention and different interventions to reach ethnic minority women, the challenges of how to compose an intervention to increase screening participation among these women continue to exist. In order to design an intervention it is essential to understand barriers seen from the intervention target groups’ perspectives. Therefore, the voices and perspectives of ethnic minority women themselves are crucial for turning the tide of social inequalities in cancer screening [20].

The aim of this study was to explore perceptions about cancer and barriers towards cancer screening participation among ethnic minority women.

## Methods

### Setting

This study was carried out in Gellerup, which is a socially deprived suburban area in Aarhus, the second biggest city in Denmark with more than 345,000 citizens [21]. Gellerup has more than 5600 citizens and is characterised by an unemployment rate of 52.5, and 79.4% of the citizens are from non-Western countries, predominantly from the Middle East and Somalia [21, 22].

The Danish medical system is based on the principle of free and equal access to preventive care and treatment for all residents. This includes free access to nationwide cancer screening programmes and any follow-up visits and treatment derived from participation in cancer screening. Since the 1960s cervical cancer screening has been gradually implemented. Today the Danish women are invited to participate in screening for cervical cancer every 3 years when aged 23–49 and every 5 years when aged 50–64. The invitation is sent by digital mail recommending the woman to book an appointment at a general practitioner to have a cervical cytology specimen collected during a gynaecological examination [23]. All Danish women aged 50–69 are since 2007 also offered biennial breast cancer screening. In the screening invitation, women are offered a pre-booked mammography appointment at a screening hub [24]. The colorectal cancer screening programme was implemented in 2014 for citizens aged 50–74 and uses faecal immunochemical test self-sample kits, sent directly to the home, with the invitation to collect a faecal sample and mail it directly to a laboratory [25]. All programmes have a reminder system. Citizens, who do not participate despite invitation and reminder(s) receive a new invitation in the next screening round if they are still in the relevant age group, unless they have actively opted out of the screening programme. All communication and written material about the cancer screening programmes is in Danish.

The Danish cervical cancer screening programme has a participation rate of 74.5% for Danish women and 61.1% for ethnic minority women [8]. The Danish breast cancer screening programme has a participation rate of 79.6% for the Danish women and 57.3% for ethnic minority women [12]. The Danish colorectal cancer screening programme has a participation rate of 71.5% for the Danish women and 56.1% for ethnic minority women [13].

### Design and recruitment

This was a qualitative interview study with ethnic minority women. The interview study was based on a phenomenological approach [26] aiming to describe the phenomenon ‘cancer screening’ as seen from the ethnic minority women’s lifeworlds. Thus, the phenomenological perspective allowed us to gain a greater insight through subjective experienced descriptions, and it served as a guiding principle for the research process.

The participants were recruited through snowball sampling [27] in different social societies in the area. Co-author TBR identified the eligible participants from her general practice surgery in Gellerup according to a maximum variation sampling strategy including ethnic minority status from non-Western countries (both first and second-generation immigrants) and ages between 23 and 74 years old [28]. TBR gave, with her patients consent, their contact information to first author CRT. In parallel with TBR’s recruitment, CRT interacted with the people in the area and contacted different communities and local cultural societies, for instance “the Neighbourhood Mothers”. This local society includes women with an ethnic minority background who do voluntary work in the community by supporting vulnerable women, to convey important information and build bridges between ethnic minority women and the Danish society [29]. CRT was introduced to the area by the Neighbourhood Mothers and recruited participants by a face to face approach. The study aimed to reflect the views of as many ethnic minorities as possible in the deprived area; therefore recruitment of participants with limited Danish language skills was relevant. With guidelines from CRT the Neighbourhood Mothers also recruited participants to the interviews. Additionally three experts in Somali, Arabic and Southeast Asian culture were recruited to individual interviews. They were members of different executive committees or handpicked based on their social engagement in the community. All eligible participants were given verbal and written information about the study in Danish or in their native language before the interviews.

### Data collection

The interviews were conducted from April to June 2019. The study included tree different types of interviews; semi-structured focus groups, group interviews with an interpreter and individual interviews with experts in different cultures. An assessment of the information power during the collection period determined the number of participants. Data was obtained until the collection of statements and perspectives were large enough to describe the phenomenon and reach the aim of the study in a satisfactory manner [30].

A semi-structured interview guide was designed to assess specific themes, while allowing the participants to communicate freely in their own words about their experiences and perceptions of cancer and screening [31]. It covered the following themes: healthcare seeking behaviour, health perceptions, knowledge about cancer, cancer prevention and early detection, knowledge about cancer screening, individual and socio-cultural barriers, attitudes towards the screening programmes and cancer screening invitations, coping strategies and facilitators to screening (see the interview guide, Table 1). In the last part of every interview a short presentation was held (15 min) by one of the researchers about cancer and screening to observe the participants’ responses to the rationale behind the Danish cancer screening programmes, and gain insight into the participants’ pre-existing knowledge about cancer screening. The participants were also introduced to a faecal immunochemical test self-sampling kit used in the Danish colorectal cancer screening programme. Furthermore they were introduced to two different types of HPV (human papillomavirus) self-sampling kits for screening for cervical cancer. These two self-sampling kits are not yet implemented in the Danish screening programme. The interview guide was based on discussions in the research team and used for the different types of interviews. The interview guide and the phrasing of the questions during the interviews were slightly modified according to the interview type and the participants’ language skills. In this study we present the results concerning perceptions and perceived barriers towards cancer and screening.

**Table 1:** Interview guide

Research questions	Themes	Questions
	Briefing	Presentation round (research team and participants) followed by a short introduction to the study, oral consent and how a focus group works.
PART ONE: Own health, cancer and the medical system		
How do ethnic minority women experience the Danish medical system?	Healthcare seeking behaviour	• How do you feel about going to your GP in Denmark? Vs. your native country? Relationship to the GP? • When and why do seek medical assistance?
Which perceptions about health do ethnic minority women have?	Health perceptions	• Do you know any health myths? What is a healthy body? Responsibility? Do you worry about your health? • Sources of knowledge?
What do ethnic minority women know about cancer?	Knowledge about cancer	• Why do we get cancer? Causes? Treatment? Sources of knowledge?
What are ethnic minority women’s attitudes toward prevention and early detection	Prevention and early detection	• Can you be sick without any symptoms? Can you go to your GP without any symptoms? What does it mean to prevent? What are the benefits with early detection?
PART TWO: Cancer screening		
How aware and what do ethnic minority women know about cancer screening?	Knowledge about cancer screening	• Do you know why you are invited to screening? Purpose? Relevant? • Are you vaccinated (HPV)?
Which specific individual and cultural barriers may prevent ethnic minority women from participate in cancer screening?	Individual and socio-cultural barriers	• What do you consider before deciding whether or not to get screened? Does the opinion of others matter to you? Language? Concerns?
	Short presentation about cancer and screening	
What are the attitudes toward preventive cancer screening?	Attitudes towards the screening programmes	• Are you more likely to participate in one program than another? Why? How do you feel about the different screening procedures?
Which specific system related barriers may prevent ethnic minority women from participate in cancer screening?	Cancer screening invitation and coping strategy	• What happens when you get invited to screening (step by step)? • What do you think about ‘e-boks’ (digital mail)? What do you use ‘e-boks’ for? • How would you like to be invited and informed about cancer screening?
Which ideas do ethnic minority women have that facilitate cancer screening?	Facilitators to screening	• How can we get cancer screening more relevant for you? • What do you see as an obstacle to get screened? • Opinions about other ideas (from other focus groups and literature)
	Debriefing	Thank you for your participation – Is there anything you would like to add?

Prior to the interviews, the participants were asked to complete a consent form and a questionnaire designed by the research team. All interviews started with a short introduction to the study. The interviews ended with a debriefing where the participants were given an extra opportunity to speak one’s mind. After the interviews, the participants received a gift voucher (25 euros) as an appreciation of their interview participation. All the interviews were audio recorded and transcribed verbatim afterwards. Depending on the participants’ wishes, the interviews took place at a closed beauty salon, a meeting room at the local mall, or a meeting room in a community house. Immediately after each interview, the interview was discussed among the researchers and notes from the interview were compared and elaborated for later use in the analysis.

The focus groups were conducted by minimum two researchers (CRT, SBE and/or PK). CRT has experience with conducting focus groups and interviewing vulnerable people. PK is an experienced interviewer and researcher using qualitative methodology. SBE is knowledgeable about ethnic minority women and a skilled medical teacher. CRT was the moderator in the interviews and PK and SBE supplemented the discussion. The constellation with more than one researcher allowed all the researchers to concentrate on listening, understanding, taking notes and asking questions.

The group interviews were conducted by CRT, PK and a tele-interpreter. The participants were asked to complete the same consent form but in a translated version in their native language. The individual interviews were conducted by CRT. Preliminary data from the focus groups and the interviews with a tele-interpreter were added to the semi-structured interview guide for the individual interviews with experts for discussion.

### Data analysis

The inductive content analysis of the interviews focused on the contextual meaning of the women’s perspectives, experiences and descriptions. The purpose was to explore the research questions (see the interview guide, Table 1) based on data instead of verifying data based on a predetermined theory. Therefore, the women’s perspectives on cancer and experiences with cancer screening evolved freely from the material rather than from established categories. As part of the preliminary analysis, the interviews were carefully read to obtain an overall impression of the material, and additionally the field notes from the interviews were used to contextualise the findings [32, 33]. The analysis began with an open coding of the material, followed by the development of subthemes and finally main themes. Initially this process was conducted by CRT, after which CRT and PK discussed the groupings of the codes to subthemes to main themes. The analysis was a dialectical interaction between the steps mentioned, before the results were final [34, 35]. The research team met regularly to discuss the analysis and results.

## Results

A total of 37 women from ten different non-Western countries participated in the study based on five semi-structured focus groups, two structured group interviews with an interpreter and three individual interviews with culture experts. Some of the interviews were homogenous with only Arab or Somali women, and others were ethnically heterogeneous (see Table 2). The length of each interview (seen Table 2) included briefing and debriefing and varied from 54 min to 130 min. Eleven participants were employed, 12 were unemployed, two were self-employed, five were enrolled in education, one was granted an early retirement and six did not state their employment status. The participants’ age ranged from 27 to 59 years old with a median of 39 years. In Table 2, years of stay in Denmark are stated in 10 years intervals, and information about the participants’ age is divided into two age categories in order to secure anonymity of the participants. All names are pseudonyms.

**Table 2:** Characteristics of study participants (*N* = 37)

Interview	Length	Name (pseudonym)	Age (years)	Origin	Years of stay in Denmark
Focus group 1	130 min	Dilara	> 39	Turkey	> 29
		Ceren	> 39	Turkey	20–29
		Lina	27–39	Iraq	20–29
		Naima	27–39	Iraq	10–19
		Idman	27–39	Somalia	20–29
Focus group 2	89 min	Amina	> 39	Lebanon	10–19
		Musa	27–39	Syria	0–9
		Aisha	> 39	Lebanon	20–29
		Malak	27–39	Lebanon	10–19
		Yasmin	27–39	Saudi Arabia	20–29
Focus group 3	118 min	Ninel	27–39	Uzbekistan	10–19
		Alina	27–39	Uzbekistan	10–19
		Emin	27–39	Turkey	> 29
		Jamal	27–39	Morocco	> 29
		Safa	> 39	Pakistan	> 29
		Anna	> 39	Uzbekistan	10–19
Focus group 4	81 min	Yusra	> 39	Lebanon	20–29
		Aida	27–39	Syria	20–29
		Tasnim	> 39	Lebanon	20–29
		Khadija	27–39	Syria	0–9
		Huda	> 39	Lebanon	20–29
		Iman	27–39	Lebanon	20–29
Focus group 5	102 min	Gobey	27–39	Somalia	20–29
		Yusur	> 39	Somalia	10–19
		Tawfiiq	> 39	Somalia	20–29
		Aaliyah	27–39	Somalia	20–29
		Weris	27–39	Somalia	10–19
		Barkhado	> 39	Somalia	20–29
Group interview 1	54 min	Isra	> 39	Syria	20–29
		Aziz	27–39	Lebanon	10–19
Group interview 2	83 min	Hibaaq	27–39	Somalia	0–9
		Farax	27–39	Somalia	0–9
		Yurub	> 39	Somalia	0–9
		Xusni	–	Somalia	–
Expert interview about Somali culture	55 min	Amaal	> 39	Somalia	10–19
Expert interview about Arab culture	77 mintues	Nouria	> 39	Morocco	> 29
Expert interview about Southeast Asian culture	62 min	Huynh	> 39	Vietnam	> 29

Four main themes were identified and explored: Knowledge about cancer and screening, inadequate language skills in Danish, social and cultural beliefs and mistrust in the Danish medical system.

### “There is no need to get screened if I don’t feel anything”: knowledge about cancer and screening

#### Perceptions and knowledge about cancer

All participants said that they knew someone who had cancer or had died of cancer, and they perceived it as a very common disease that everyone can get, like Nouria explained it: “*It’s [cancer] becoming more and more like a flu epidemic. So, it gets much more normal to hear about people who have cancer*”. Many participants reported that unhealthy diet, lack of exercise, stress, smoking and genes that run in the family might contribute to developing cancer. Only a few participants mentioned that these factors were less important because in the end, cancer was the will of God.

Many of the participants reported that they did not get screened because they were afraid of knowing that they had cancer. Some said that they preferred to die without knowing they had cancer, like Idman put it: "*The thing is that I don’t know if it’s conscious or unconscious, but I also kind of fear "what if they find something?". And another thing I keep thinking about is that I don’t feel any pain, I don’t have any symptoms that make me think it needs to be examined"*. The fear of cancer emanated from the perception that cancer is not curable and getting diagnosed with cancer was like getting a death sentence for many of the participants. Emin expressed it this way: *"I don’t want to say this out loud; but I think that I feel that you can’t survive cancer. Now I said it [laughs]."*.

#### Unfamiliar with prevention and early detection of disease

The participants showed fragmented knowledge about cancer screening. Most of the participants had heard about screening before but did not know the purpose of it and therefore had little awareness about the screening programmes. Some of them were unsure whether they had attended screening in the past. Nearly all the women had the perception that screening should only be performed on people who had symptoms. Naima explained her view on screening like this: *"I think I was invited to get screened twice, but I didn’t go [laugh]. I threw out all the papers. There is no need to get screened if I don’t feel anything."*. Overall they only sought medical services when they experienced symptoms.

The participants came from countries with different health care systems where population-based cancer screening programmes were not implemented or were not free of charge. The health care strategy with early detection and prevention of cancer was for most of the participants difficult to understand and talk about. An example of that was the conversation between Idman and Dilara:

*I: Cancer I think is sometimes more abstract. After all, it can occur almost anywhere. The fact that you can prevent cancer is a little difficult to understand*

*D: It sounds like a myth*

*I: Yes, that it can be prevented?*

We found the participant’s interest in health issues to be variable. Some said that they talked a lot about it and others said that they did not discuss it with each other, like Amaal said: " *… It’s not like you talk a lot about health. [ …*] *not many go to a doctor or to the hospital – it’s like an unknown place. Many think 'If I die, then I die' "*. Still all the participants wanted to know more about cancer and prevention during the interview. Ceren talked about how screening was new to her: *"I don’t know anything about the subject because in Turkey it is very taboo to talk about it [cervical cancer and sex]. We lack knowledge about many things"*. During the interviews all the participants showed interest in the cancer screening programmes and had very curious and positive attitudes toward them. They showed their curiosity by asking a lot of questions about cancer and screening during the interviews. Furthermore, many of them were distracted by their cell phones in the beginning of the interview sessions, but this behavior eased off quickly. The positive attitudes came to show by positive responses to the short presentation held during the interviews by one of the researchers about the rationale behind the Danish cancer screening programmes. Many had an aha experience and admittedly said that they were thankful for their new insights about the subject.

### “If it is only in Danish no one looks at it”: inadequate language skills in Danish

#### Information in Danish only

Many of the participants mentioned that ethnic minorities in Gellerup did not speak and understand Danish well enough. Huda put it this way: "*Over half of people living here can’t read at all. They may understand, but they can’t respond to it"*. Jamal described how she found the written material strange and unfamiliar: *"... So all the material you get you don’t read it because you don’t understand it … If it is only in Danish, no one looks at it*". This was the main reason for her not to read it. The rest of the participants in the focus group agreed to this perspective.

#### Verbal culture and information primarily from native country

The participants reported that the Arab and Somali communities in Denmark are verbal cultures. They primarily got their information about health from their native countries and social media. Aisha mentioned: "*Everyone is on Facebook. I’m really good at Facebook"*. Tasmin told how they were kept up to date by each other on many different subjects: "*We talk about it [health] a lot. Whenever we hear something new we pass it on [laughs]"*. They preferred to use each other for medical advice rather than seek medical care. Nouria expressed it this way: "*There is always someone who has the same symptoms and has tried different treatment methods. So you think ‘maybe I should try that’. Then you can sort of feel your way forward like that".*

### “I mean, when you are a virgin, you are not examined “down there””: social and cultural beliefs

#### Circumcision and cultural beliefs about virginity

The Somali and Arabic women were sceptical about participating in cervical cancer screening before marriage, but for two different reasons: The Arabic women expressed concern that the screening procedure itself would jeopardise their own or their daughters’ virginity. Aida said: *"You do not do genital examinations on girls who are not married because if something happens and she is not a virgin anymore, it can have many consequences, right?"*. The Somali women in the study reported that female circumcision was a barrier for cervical cancer screening. Idman explained it like this: *"The circumcision is the reason why we don’t participate. You choose not to come to an examination before you are married. [Before marriage] you are circumcised and sewn together, so a physical examination is not possible... And most young people who are not married they choose to not get the screening for that reason"*.

After marriage, however, most of the Somali and Arabic participants said that they were open for participating in the screening programmes. Many described the thought of a gynaecological examination or mammography as embarrassing, uncomfortable or painful but were at the same time willing to have it done. Some mentioned that it was easier getting a gynaecological examination after they had given birth and described it as a quick and painless procedure. Only three participants would refuse the offer – one because she was traumatised from her circumcision and the others because they could not see the purpose of it.

#### The older generation

Younger and older participants had different attitudes and knowledge about cancer and screening. Khadija explained the different attitudes with an episode she had with her aunt: "*My aunt for instance – she got an invitation for breast cancer screening. So, I said to her "Aunty, you got an invitation", "Why should I?"[laughs]. Then I told her "Come on, It’s a really nice offer""*. When the participants were asked how their parents, husbands or other family members would react on a screening invitation, most of the participants laughed because it was hard for them to imagine them participating in cancer screening. Lina was one of the participants laughing and said: "*Sending their faeces may be transgressive for the older generation. They can’t understand why researchers want to mess with it [everyone laughs]"*. Some had mothers who did not trust the screening programmes and thought that the test itself would infect them with cancer. Therefore some of the participants were recommended not to participate in screening by their parents. Others from the older generation were very happy about the screening programmes. The oldest participant Aisha put it this way: "*I always go for examination, for screening. I have never forgotten one. Neither cervix nor breast. I go for all of them"*.

### “We don’t really trust the Danish system”: mistrust in the Danish medical system

#### Doctor-patient relationship

There was large variation in the participants’ views on the Danish medical system. Many participants were content with their own general practitioner and felt that they had a good relation with each other. Ceren told how she preferred her Danish general practitioner over the Turkish doctors in her native country. She expressed it like this: “… *The Turkish doctors give me like 10,000 kinds of medicine. I hate it. I call Denmark to talk to my own general practitioner”*. At the same time many criticised their doctor for having long waiting time, problems with booking time, and treatments based on watchful waiting, drinking water or over-the-counter painkillers. Safa put it like this: “ … *The famous Panodil [paracetamol, pain killer] that helps with everything. […*] *you can put out a fire, but embers are still there”*. In situations with serious diseases many mentioned that doctors had involved them in the decision-making process about their own or close-related family member’s treatment. They found this strategy strange because according to them it was the doctor’s responsibility to pick the right treatment for the patient. Furthermore, nearly every participant had experienced how the Danish doctor used “Google” during their consultation. This made some of them think less of the Danish doctors, like Naima said: “*… Here the [Danish] doctors have zero IQ”*.

#### Second opinion

Most of the participants expressed mistrust towards treatment at the hospital and the doctors at the hospital. Nearly all the participants had experienced misdiagnosis or heard stories about it. Therefore many preferred a second opinion in Germany or in their native country. Aaliyah explained it this way: “*If I have to be 100% honest with you; I don’t think there is trust in the [Danish] doctors. There are a lot of people like me who questions their diagnosis and travel to Germany for a second opinion*”. In some of the ethnic minority communities, German doctors had a high reputation.

## Discussion

The purpose of this study was to gather knowledge to better understand ethnic minority women’s perceptions about cancer and perceived barriers towards cancer screening. The most widely held perceptions among the women were that screening was only relevant for women with symptoms and therefore they had low awareness about cancer screening. At the same time they were very interested in the subject and wanted to know more about cancer and screening. The most dominant barriers in the accounts of the participants were fragmented knowledge and inadequate language skills.

### Strengths and limitations

The sample was recruited through snowball sampling. In general participants often refer those whom they know and have similar traits with. This can be a potential limitation with the method [27]. We were aware about this disadvantage of snowball sampling, and therefore participants were recruited from different communities in the deprived area. We did not have women resign from participation, but sometimes more people showed up for the interview than expected. They were all eligible and thus allowed to participate. This indicated a successful strategy for recruitment of a potential vulnerable group. Other sampling methods may not have been able to provide these sensitive samples.

The variation in the sample was considered a strength to the study. The sample was a highly diverse group regarding their ethnicity, age, years of stay in Denmark, and occupation. This may have increased the representativeness of the information provided by the women. We varied the ethnic composition of the focus groups. Some focus groups were very homogenous with only Arabic or Somali women, and the others were more ethnically heterogeneous. Both compositions lead to a great dialogue and discussion. Some of the participants had a dominant personality, while others were limited by their inadequate Danish language skills - which sometimes made it difficult to moderate the focus group. This was handled by explaining that we were interested in getting as many different points of view as possible and asking the more quiet participants directly during the interview.

The findings in our study may be transferable to other ethnic minority women in deprived areas in a healthcare system which is publicly funded by taxation.

### Interpretation of results

Emotional responses to screening like fear, pain or discomfort and embarrassment were mentioned by many of the women. These emotions did not, however, appear to be the most crucial barriers for screening participation. Fragmented knowledge about cancer screening and inadequate language skills were more prominent barriers among the participants. Previous quantitative studies also showed lower awareness of cancer warning signs and screening programmes among ethnic minorities [5, 36, 37].

Studies from other countries have confirmed that minority women have inadequate language skills which may hinder participation in screening. A study from 2017 [38] found language to be one of the dominant barriers to cervical cancer screening in Norway. Another study from 2015 [39] sought to explore reasons for lack of participation in colorectal cancer screening in England among South Asian minority communities. The study found that communication via written materials came across as being particularly inappropriate for the Muslim community due to the largely verbal culture of this community. Other studies found that language barriers undermined both the access and the quality of health services for immigrants [40, 41].

The results in our study showed cultural differences in perceptions about health care professionals. Many of the participants were not familiar with being a part of the decision-making process during the doctor visit. They explained how they were used to a paternalistic attitude from the doctors from their native countries. The participants experienced the Danish doctors to be more egalitarian and involved them in the decision-making process. This made some of the participants doubt the Danish doctors’ professional competencies, while others preferred the Danish way. This is confirmed by Napier et al. who claimed that: “*the systematic neglect of culture in health and healthcare is the single biggest barrier to the advancement of the highest standard of health worldwide*” [42]. The patient and the doctor communication could be hindered by cultural differences [42, 43]. This could potentially lead to a lower quality of care [44]. A high-quality delivering of patient care depends largely on the communication between patients and doctors [45].

This study adds to the existing literature with a new perspective. Other literature found that cancer was described as stigmatised. Studies from Norway and USA [38, 46] found that cancer evoked secrecy and shame for ethnic minority women. The participants in our study had the perception that everyone could get cancer and they did not describe it as stigmatised. On the contrary they showed great care for those whom they knew had cancer. Some of the participants were more religious than others in this study and had fatalistic views. The religious participants believed that it was God’s will if cancer was present. This is also reflected in the literature [47, 48]. Despite their religious beliefs it was acceptable to participate in screening and they were open to the idea. This in accordance with a study from 2014 [46] that found that religion might not be a prominent barrier towards screening participation.

The study’s findings indicated the potential and the need to develop a culturally adapted and tailored intervention to increase ethnic minority women’s knowledge and awareness about cancer screening. The different barriers for participating in screening are potentially modifiable with the right culturally adapted and tailored intervention based on different strategies such as educational, linguistic and awareness raising programmes.

### Conclusion

The ethnic minority women in the study believed that cancer screening was only relevant if they had symptoms. They had fragmented knowledge about cancer screening and cancer prevention. Furthermore, they had inadequate language skills in Danish which made it difficult for them to interact with the Danish medical system. Attitudes to cancer screening were characterised by openness and the study showed positive and curious attitudes towards the offer to get screened. The verbal cultures among the participants and their networks were strong. They were an important source of information to each other. The insight from this study could serve as a basis for an intervention to increase cervical, breast and colorectal cancer screening among ethnic minority women. Men are also invited to colorectal cancer screening, but their perspectives were only captured by the women’s statements in this study. Further research should include an investigation of ethnic minority men’s perceptions about cancer and barriers towards cancer screening.

## Data Availability

The datasets used and/or analyzed during the current study are available from the corresponding author on reasonable request.
